# Differential Symptoms of Epidemic Sore Throat and Diphtheria

**Published:** 1875-10

**Authors:** A. L. Knight

**Affiliations:** West Columbia, West Virginia


VOL. V.—OCTOBER, 1875.—NO. 10.

### Original Communications.

### DIFFERENTIAL SYMPTOMS OF EPIDEMIC SORE THROAT AND DIPHTHERIA.

*Read before the Meigs County (Ohio) Medical Society, August* 16, 1875.

*Mr. President and Gentlemen:*

Before entering into the differential symptoms of these two diseased conditions, I shall offer some remarks upon the pathological state and appearances of inflamed mucous and epithelial tissues in general.

Acute catarrh, or catarrhal inflammation or irritation, of the air passages, including the throat, covers all inflammations of those parts not specific in their nature. That is to say, that any irritation or inflammatory action in these organs, not having a specific cause, are, by common consent, referred to catarrhal influence. Had we any expectation of a solution to the question, we might ask, with propriety, what is catarrh? But our ignorance of its nature does not debar us of its phenomena or the essential symptoms that universally attend. We know of various theories regarding its cause, but as none of them point to any particular factor, or even factors, we must content ourselves, at least for the time, with its phenomena.

Charles Ewald Hasse, formerly Professor of Pathology in the

University of Zurich, says: "Catarrh is a disease of every-day occurrence, and spares neither age nor sex; is fraught with but little danger, unless where widely diffused over the mucous membrane, or seated in the majority of the minute bronchial ramifications, or where the individual is deficient in vital reaction, either by nature or disease.

"Acute catarrhal irritation of the mucous membrane is characterized at the commencement by irregular rosy patches, nowhere distinctly circumscribed, but as if shaded off at their circumference. This reddening is occasioned by fullness of the extremely delicate superficial vessels, which, however, even in this condition, still admit of artificial injection. In a short time, however, the above patches exhibit scattered vermillion points, whilst the pale, red, vascular network becomes darker and appears to go to a greater depth. The vermillion presently become more numerous, unite to irregular, undulating streaks, and ultimately coalesce in patches over the entire surface, imparting to the mucous membrane an uniform tinge, and changing its thin, smooth aspect into one resembling plush. A ramifying of blood-vessels is now no longer visible upon the surface, though very evident in the submucous tissue. The upper layer of the mucous membrane is mostly dry, double its natural thickness, and easily torn. It will be noticed that the term irritation is used as an exposé of the above conditions." How far they can be considered removed from inflammation of those parts, I leave to your decision. My own opinion favors the idea that they indicate incipient inflammation. Even the author just quoted says that these irritations may run into inflammation with destruction of tissue.

I do not propose to theorize upon the pathological conditions of irritation and inflammation, but wish to draw attention to some physical appearances common to both, regardless of causes inducing them. It is well known, says Hasse, "that in the healthy state, the epithelial investment is being continually, though imperceptibly, cast off, and regenerated out of the secretion proceeding from the true surface of the mucous membrane."

Now, in a congested state, the tesselated or plaster epithelium, according to the same author, consists of various layers of epithelium-cells in a more or less advanced state of development from without inwards. The changes produced by catarrh (it seems conclusively that he might have stated from this condition of the parts, from whatever cause), consist essentially in the following:

"Between the true surface of the mucous membrane and the epithelium, an effusion takes place, at first almost exclusively of serum, by which the epithelial covering is raised, and here and there allowing the escape of watery effusion. After some time, small, isolated groups of cyliary cylinders become separated, and, ultimately, the whole coating is either removed all at once, or dissolved, rolled into pellets and thus rejected."

Thus it will be perceived that certain unknown causes, acting on the system, perhaps upon the nervous, producing congestion of the superficial capillaries, with no material change in the finer arterial structures, or other signs of inflammation being present, can and does produce pellicular exudations, with tumefaction of the parts in which it is confined—added to which is the frequent transformation of mucus into true pus globules—known by the dark colored nuclei, readily seen, when treated with acetic acid, by a microscope of low power. Hence, how easily we might confound these conditions, taking this one simple physical sign, common also to inflammatory action, with diseased action, brought on by entirely different agents.

Yet, let it be borne in mind that a mistake of this kind is extremely unfortunate. The indications of the plans of treatment are so diametrically opposite. When the above conditions are the result of local inflammation, they readily yield to antiflagistic remedies, which are decidedly prejudicial in cases having a different origin. To diagnose in complications, just alluded to, is often very difficult, especially where the irritation is assuming a transition into what may be styled an inflammatory stage, from a repetition of attacks of irritation, and perhaps having new factors entering as collaterals during the progress of the original distemper.

This brings us to the examination of simple inflammation of the throat, pharyngitis, or epidemic sore throat, or vernal sore throat, appearing throughout our entire country, in each successive year, during the changeable weather of early spring, and not unfrequently appearing epidemically in that season of the year. The inflammation, though confined to the throat, generally, may attack the tonsils and posterior nares; when confined to the pharynx, the symptoms are, at first, dryness of the throat, with frequent and painful attempts to swallow; the dryness is succeeded by increased watery mucus, which often relieves. If not thus relieved, there is thrown out plastic lymph, which is more or less adherent to the mucous membrane, and becoming blended with the epithelium, form pellicular exudations that resemble diphtheria, and at this stage may be detached, though with difficulty, from the subjacent mucous tissue. This is the more difficult, when the exudations are thrown out within the mucous folicles of the tonsils; but as a rule these pellicular films are easily broken up. Any hard substance brought in contact with them tears them, even when they cannot be entirely detached, and this is one, though perhaps a minor differential symptom in the two affections in question. This inflammatory affection, when it descends to the inferior part of the pharynx, and merges upon the larynx, is called by the English and American authors, angina membranica, some times extending into the larynx, and trachea, inducing that slow and insidious form of croup, (so much dreaded by every practitioner of medicine), which is a modified but aggravated form, contrasted with that of irritation and congestion, from the effects of simple catarrh. (See Hasse, Art., Catarrh of the Air Passages.)

A fuller description of inflammation of the throat, with its complications with epidemic catarrh or influenza, would transcend the limited purpose of this paper; but before dismissing this part of the subject, I will say that, however grave the symptoms may appear in this form of sore throat, growing out of inflammatory action, the prognosis is always favorable. I will venture the assertion that every uncomplicated case will recover with the ordinary antiphlogistic remedies, and often without any remedies at all. Of course, I mean by antiphlogistics, both local and general remedies, including topical applications, cold or hot, as the case may indicate, purgatives, scarifications, sudorifics, etc.

And further, gentlemen, it is in these cases, in this special kind of *diphtheria,* that many wreath their brows with laurels from victories quite easily won; whilst those who call it by its proper name, pharyngitis, and treat it as such, and relieve it as such, are, as a rule, not very *good* doctors in *diphtheria.* So I have heard laymen say, at least. But, say they, what shall we call it, there is a diphtheritic membrane, why not call it diphtheria? I should have no objection to the name, were it not that angina maligna is also known by the same name, a disease purely asthenic in character, having the diphtheritic membrane, in common with sthernic membranous sore throat.

It is nevertheless true that, in tl.e years 1818 to 1821, both forms of the disease appeared in France, particularly at Tours, and was described by Brettonneau, who made no distinction between them, except the differing forms of the accompanying fever, gave the name diphtherite to each; hence came the cognomen of "croup diphtherite." Dunglison says, (Cyclopædia of Practical Medicine, art., Throat Disease,) that "in the angina maligna there is, from the beginning, great prostration and disorder of the nervous system; the pulse is weak, though not very rapid, and the skin moderately warm, or even cool. There is at first *not* much apparent affection of the throat, but the act of deglutition is very soon observed to be performed with great difficulty, so that liquids are often returned through the nostrils, which cannot be accounted for by the trivial degree of swelling of the throat; the submaxillary lymphatics become swollen and painful, the breathing hurried and laborious, now and then there is a threatening of suffocation, and an accrimonious secretion, mixed with portions of false membrane, discharged from the nose. When these membranes are tinged with blood, and especially when at the same time blood exudes from the lips and gums, their dark or almost black appearence, and intolerable fetor, have frequently caused them to be mistaken for sloughs. The surface, however, from which they are detached is perfectly entire; the mucous membrane is not even softened, nor is there the peculiar odor of gangrene."

The above is a good description of true diphtheria, and there is reason to doubt that there is any inflammation whatever connected with the affection. Neither the heat of skin nor the arterial excitation indicates it; nor the exudation, as described, favor our ideas of inflammation. It is not the usual product of such a condition, yet most writers have held a contrary view. Brettonneau simply made this a species of the same class of disease. Query: How do you treat this form of diphtheria? With antiphlogistics? Certainly not. Some have taken the ground that diphtheria, in the badly fed and broken down subjects, always takes on this form. This is simply false. We never fail to have that class of persons; we have pellicular sore throat annually, why do we then not see more of the graver forms of diphtheria? No, gentlemen, it is simply absurd.

Diphtheria, as now understood, is an epidemic disease, appearing at long intervals, infectious and specific in character. Persons who get through it are not obnoxious to a second attack. It is a form of typhus, not yet clearly defined, or its real cause known, and I venture the assertion that there has not been a case of it seen in this section for twelve years. We had a mild epidemic of it in this valley in 1841, and a graver form of it in 1858 to 1861, and it is to be hoped that years may elapse before it again returns.

Very Respectfully,

A. L. Knight, M. D.

*West Columbia, West Virginia.*

